# ﻿Unveiling the identity of *Diaurora* Cockerell, 1903 (Bivalvia, Unionidae): morphology, molecular phylogenetics, and the description of a new species

**DOI:** 10.3897/zookeys.1173.106148

**Published:** 2023-08-03

**Authors:** Zhong-Guang Chen, Yu-Ting Dai, Shan Ouyang, Xiao-Chen Huang, Xiao-Ping Wu

**Affiliations:** 1 School of Life Sciences, Nanchang University, Nanchang 330031, China Nanchang University Nanchang China

**Keywords:** Changjiang River, freshwater mussel, morphology, phylogenetic analysis, taxonomy

## Abstract

The monotypic freshwater mussel genus *Diaurora* Cockerell, 1903 has long been enigmatic due to its rarity and morphological confusion with *Acuticosta*. In this study, we comprehensively redescribed *Diauroraaurorea* (Heude, 1883) through a detailed analysis of shell morphology and molecular phylogenetics of recently collected specimens. Moreover, a new species, *Diauroralaeve***sp. nov.**, was identified from the Fuyishui River, a tributary of the Zishui River in Shaoyang County, Shaoyang City, Hunan Province, China. Molecular phylogenetic analyses showed that *D.aurorea* and *D.laeve***sp. nov.** were reciprocally monophyletic and formed a clade as sister to *Schistodesmus*. Our study underscores the necessity of further exploring the diversity of freshwater mussels in understudied small tributaries throughout China.

## ﻿Introduction

Freshwater mussels, an important component of freshwater ecosystems (Liu et al. 1979; Vaughn and Hoellein 2018; Lopes-Lima et al. 2021), represent a diverse group of bivalve mollusks that are often identified by their unique shell morphology (Lopes-Lima et al. 2017; Chernyshev et al. 2020). However, reliance on morphological characteristics for taxonomy has resulted in some confusion within the group, as some species may share similar morphological characteristics and exhibit phenotypic plasticity (Huang et al. 2018b; Wu et al. 2020; Wu et al. 2022). One such example is *Diaurora* Cockerell, 1903, a monotypic genus of small-sized freshwater mussels that are endemic to the middle and lower reaches of the Changjiang River, also known as the Yangtze River (Heude 1883; Simpson 1900; Tchang and Li 1965; Tchang et al. 1965; Wu et al. 2000; Shu et al. 2009; He and Zhuang 2013; Guo 2022).

Initially, Simpson (1900) described the genus as a subgenus of *Parreysia* Conrad, 1853, based on the original description of *Unioauroreus* Heude, 1883, and later renamed as *Diaurora* by Cockerell (1903). *Unioauroreus* was originally collected and described by Heude (1883) based on the holotype USNM 472411 (designated by monotype) from Ning-kouo hien in China. However, only a few additional specimens have been reported sporadically since its original description, rendering this species little-known and enigmatic for over a century. Due to the limited availability of specimens for study and the potential confusion arising from its easily confused morphology with *Acuticosta* Simpson, 1900, the validity and systematic status of *Diaurora* have remained controversial for an extended period. It has been treated as a separate genus (Haas 1924), a subgenus of *Parreysia* Conrad, 1853 (Simpson 1900), or even a synonym of *Acuticosta* (Wu et al. 1999).

To address these taxonomic uncertainties, we analyzed the validity and systematic status of *Diaurora* using a combination of shell morphology and molecular phylogeny. Furthermore, we redescribed the enigmatic species *Diauroraaurorea* and described a new species, *Diauroralaeve* sp. nov., from the Zishui River of Hunan Province, China. Our study sheds light on the distribution, ecology, and protection of this distinct genus, and highlights the possibility of small Chinese tributaries harboring unique freshwater mussels.

## ﻿Material and methods

### ﻿Specimen collection and identification

Specimens were collected by hand net from the tributaries of the Changjiang River and the Jiulongjiang River from 2003–2022. These specimens were fixed in 95% ethanol, and some specimens were dissected for observation of the internal structure. Measurements were taken point-to-point with digital calipers recorded to the nearest 0.1 mm.

### ﻿Molecular phylogenetic analyses

Genomic DNA was extracted from somatic tissues preserved in 95% ethanol using a TIANamp Marine Animals DNA Kit (Tiangen Biotech, China). The quality and concentration of the DNA were checked on 1% agarose gel electrophoresis and NanoDrop 2000 (Thermo Scientific, USA). F-type mitochondrial COI sequences, which are considered the barcode marker, were amplified using primers LCO22me2 (GGTCAACAAAYCATAARGATATTGG) and HCO700dy2 (TCAGGGTGACCAAAAAAYCA) (Walker et al. 2007). Polymerase chain reaction (PCR) amplifications of COI were performed in a final 25 μL volume mixture containing 1 μL of template DNA, 1 μL of each pair of primers, 12.5 μL of Green Taq Mix (Vazyme, China), and 9.5 μL ddH_2_O. Thermal cycling began with one cycle at 98 °C for 10 s, followed by 35 cycles of denaturation at 94 °C for 1 min, 50 °C for 1 min, and 72 °C for 1 min, with a final extension step at 72 °C for 7 min. The PCR products were purified and sequenced using an ABI 3730XL analyzer by Sangon Biotech (China). The accession numbers of all newly obtained sequences are given in Table 1.

**Table 1.** GenBank accession numbers of sequences used in this study. * indicates the sequences newly obtained in this study.

Species	Locality	Accession number	References
Unioninae Rafinesque, 1820			
*Diauroraaurorea* (Heude, 1883) 1	Ji’an, Jiangxi, China	OQ829360*	This study
*Diauroraaurorea* (Heude, 1883) 2	Ji’an, Jiangxi, China	OQ829361*	
*Diauroraaurorea* (Heude, 1883) 3	Ji’an, Jiangxi, China	OQ829362*	
*Diauroraaurorea* (Heude, 1883) 4	Fuzhou, Jiangxi, China	OQ829366*	
*Diauroralaeve* Chen, Dai, Huang & Wu, sp. nov. 1	Shaoyang, Hunan, China	OQ829363*	
*Diauroralaeve* Chen, Dai, Huang & Wu, sp. nov. 2	Shaoyang, Hunan, China	OQ829364*	
*Diauroralaeve* Chen, Dai, Huang & Wu, sp. nov. 3	Shaoyang, Hunan, China	OQ829365*	
*Acuticostachinensis* (Lea, 1868)	Jiangxi, China	MG462921	Huang et al. 2018a
*Schistodesmuslampreyanus* (Baird & Adams, 1867)	Jiangxi, China	MG463037	
*Schistodesmusspinosus* (Simpson, 1900)	Jiangxi, China	MG463046	
*Schistodesmus* sp.	Hunan, China	MG463043	
*Uniopictorum* (Linnaeus, 1758)	Europe	KC429109	Sharma et al. 2013
*Uniocrassus* Philipsson, 1788	Poland	KY290446	Burzynski et al. 2017
*Tchangsinaiapiscicula* (Heude, 1874)	Jiangxi, China	MG462977	Huang et al. 2018a
*Cuneopsisceltiformis* (Heude, 1874)	Jiangxi, China	MG462964	
*Cuneopsisheudei* (Heude, 1874)	Jiangxi, China	MG462970	
*Aculamprotulafibrosa* (Heude, 1877)	Jiangxi, China	MG462909	
*Nodulariadouglasiae* (Gray, 1833)	China	KX822653	Lopes-Lima et al. 2017
*Nodulariabreviconcha* Lee, Kim, Bogan & Kondo, 2020	South Korea	MT020662	Lopes-Lima et al. 2020
*Inversiunioyanagawensis* (Kondo, 1982)	Japan	MT020654	
*Inversiunioreinianus* (Kobelt, 1879)	Japan	MT020657	
*Pseudocuneopsissichuanensis* Huang, Dai, Chen & Wu, 2022	Sichuan, China	MZ540966	Wu et al. 2022
*Pseudocuneopsiscapitata* (Heude, 1874)	Anhui, China	NC042469	Wu et al. 2019
*Alasmidontamarginata* Say, 1818	USA	AF156502	Graf and O’Foighil 2020
*Lasmigonacompressa* (Lea, 1829)	USA	AF156503	
*Anodontaanatina* (Linnaeus, 1758)	Russia	KX822632	Lopes-Lima et al. 2017
*Pseudanodontacomplanata* (Rossmässler, 1835)	Ukraine	KX822661	
*Lanceolariagladiola* (Heude, 1877)	Jiangxi, China	KY067441	Unpublished
*Lanceolariaoxyrhyncha* (Martens, 1861)	Japan	MT020648	Lopes-Lima et al. 2020
*Cristariaplicata* (Leach, 1814)	Jiangxi, China	MG462956	Huang et al. 2018a
*Lepidodesmalanguilati* (Heude, 1874)	Jiangxi, China	MG463015	
*Sinanodontawoodiana* (Lea, 1834)	China	KX822668	Lopes-Lima et al. 2017
*Beringianaberingiana* (Middendorff, 1851)	Japan	MT020557	Lopes-Lima et al. 2020
*Pletholophustenuis* (Gray, 1833)	Vietnam	KX822658	Lopes-Lima et al. 2017
*Aneminaarcaeformis* (Heude, 1877)	Jiangxi, China	MG462936	Huang et al. 2018a
*Amuranodontakijaensis* Moskvicheva, 1973	Russia	MK574204	Bolotov et al. 2020
Parreysiinae Henderson, 1935			
*Coelaturaaegyptiaca* (Cailliaud, 1823)	Egypt	KJ081162	Graf et al. 2014
*Indonaiaandersoniana* (Nevill, 1877)	Myanmar	MF352275	Bolotov et al. 2017
*Parreysianagpoorensis* (Lea, 1860)	India	JQ861229	Unpublished
Gonideinae Ortmann, 1916			
*Pronodulariajapanensis* (Lea, 1859)	Japan	LC505454	Fukata and Iigo 2020
*Lamprotulaleaii* (Gray, 1833)	Jiangxi, China	MG462996	Huang et al. 2018a
Ambleminae Rafinesque, 1820			
*Lampsilissiliquoidea* (Barnes, 1823)	USA	MH560773	Unpublished
*Quadrulaquadrula* (Rafinesque, 1820)	USA	HM230409	
Margaritiferidae Henderson, 1929			
*Margaritiferadahurica* (Middendorff, 1850)	Russia	KJ161516	Bolotov et al. 2015
*Gibbosularochechouartii* (Heude, 1875)	Jiangxi, China	MG463022	Huang et al. 2018a

Sequences were aligned using MEGA v. 6.0 (Tamura et al. 2013) and checked by eye. The genetic distance, based on the uncorrected *p*-distance model, was calculated using MEGA v. 6.0. Phylogenetic relationships were reconstructed using maximum likelihood (ML) and Bayesian inference (BI). Thirty-six reliable unionid sequences from GenBank were use for phylogenetic analysis. *Margaritiferadahurica* (Middendorff, 1850) and *Gibbosularochechouartii* (Heude, 1875) were used as the outgroup for rooting the tree. ML analyses were performed in IQ-TREE v. 1.6.12 (Minh et al. 2013) with 1000 reiterations. The most appropriate model of sequence evolution (TIM3+I+G) was selected under PartitonFinder2 v. 1.1 (Robert et al. 2017). Bayesian inference (BI) was conducted in MrBayes v. 3.2.6 (Ronquist et al. 2012). The most appropriate model of sequence evolution (GTR+I+G) was selected under ModelFinder (Subha et al. 2017). Four simultaneous runs with four independent Markov Chain Monte Carlo (MCMC) were implemented for 10 million generations, and trees were sampled every 1000 generations with a burn-in of 25%. The convergence was checked with the average standard deviation of split frequencies <0.01 and the potential scale reduction factor (PSRF) ~1.

### ﻿Institutional abbreviations

**NCU** Nanchang University (Nanchang, Jiangxi, China)

**USNM**United States National Museum of Nature History (Washington, DC, USA)

**NZMC** National Zoological Museum of China (Beijing, China)

**SMF**Senckenberg Forschungsinstitut und Naturmuseum (Frankfurt am Main, Germany)

**YHC** Collection of Mr Hao Yang (Xiamen, Fujian, China)

## ﻿Results

### ﻿Phylogenetic relationships

**Figure 1.**
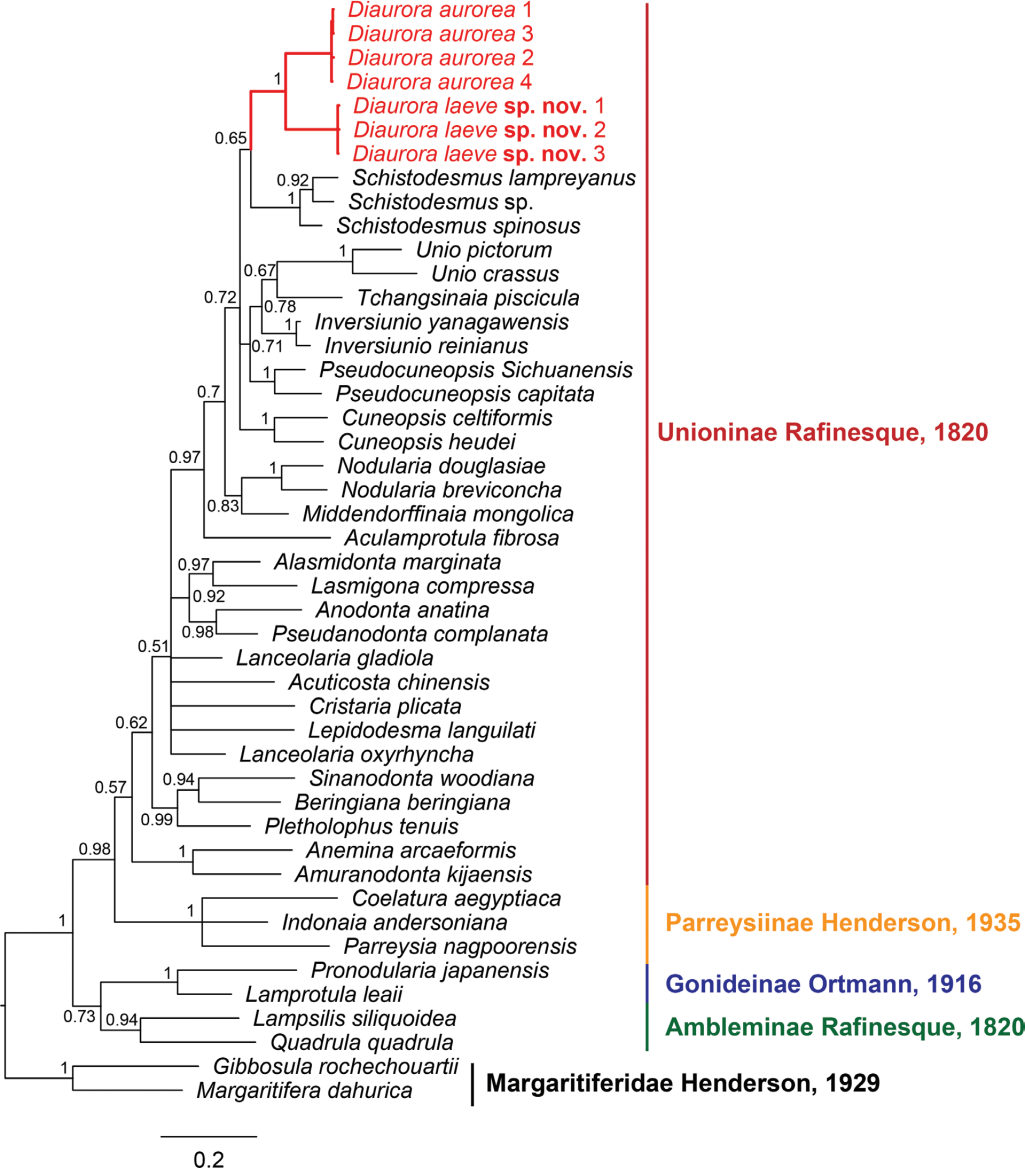
Bayesian inference (BI) tree inferred from COI gene sequences. Posterior probabilities are shown on the left of nodes on the tree if greater than 50%. Red font indicates the species from this study.

**Figure 2.**
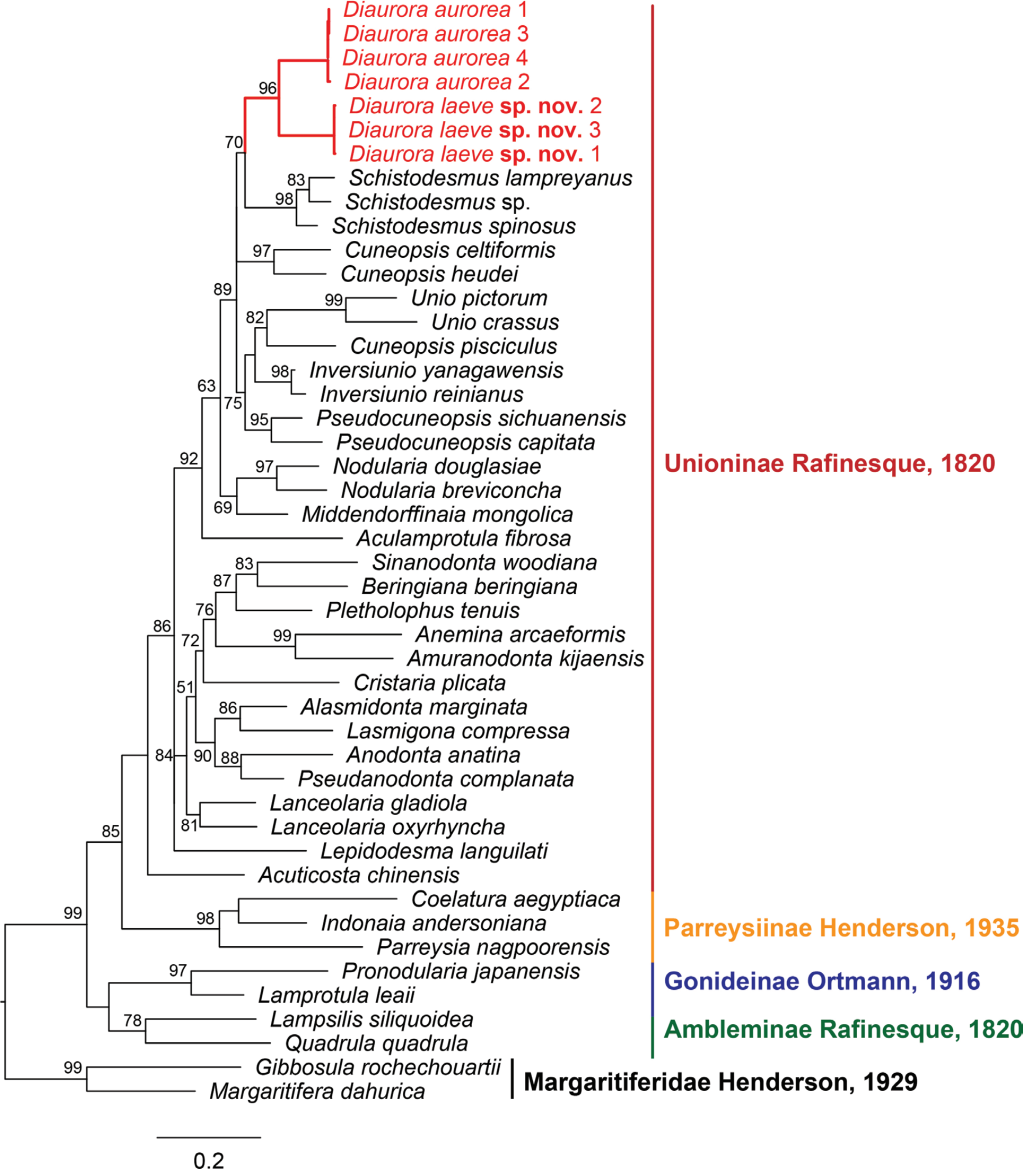
Maximum likelihood (ML) tree inferred from COI gene sequences. Bootstrap supports are shown on the left of nodes on the tree if greater than 50%. Red font indicates the species from this study.

A total of 43 mitochondrial COI sequences from 38 unionoid species were utilized in the phylogenetic analyses (Table. 1). Phylogenetic analyses revealed ML and BI trees with largely consistent topologies (Figs 1, 2). Our results showed that *Diaurora* occupied a distinct position in the subfamily Unioninae and was the sister group of *Schistodesmus* Simpson, 1900 instead of *Acuticosta*. However, this sister relationship between *Diaurora* and *Schistodesmus* was not well-supported (bootstrap support value = 70 and Bayesian inference posterior probability support value = 0.65). Furthermore, *Diaurora* can be divided into two separate clades: one consisted of *Diauroraaurorea*, and the other represents a yet-to-be-described species with distinct morphological characters. The genetic distance (uncorrected *p*-distance) between the two clades in *Diaurora* was 10.4%. Indeed, we acknowledged that deep relationships in our phylogenetic trees based on the COI dataset were not well resolved but should not overturn the main conclusion drawn here. It is necessary to further reveal the exact phylogenetic relationships using more data such as mitochondrial genomes in the future.

### ﻿Systematics

**Family Unionidae Rafinesque, 1820**

**Subfamily Unioninae Rafinesque, 1820**

#### ﻿Genus Diaurora

Taxon classificationAnimaliaUnionidaUnionidae

Cockerell, 1903

B36C7A0D-F273-5C81-8CCC-0ECD2244C93C

Figs 3A, B , 4 , 5

##### Type species.

*Unioauroreus* Heude, 1883, by original designation.

##### Diagnosis.

Shell small size, symmetric, flat, sub-glossy, triangular-ovate to reniform, orangish to brownish, with broken blackish-green rays. Zigzag sculpture present. Posterior ridge absent.

##### Remarks.

**Figure 3.**
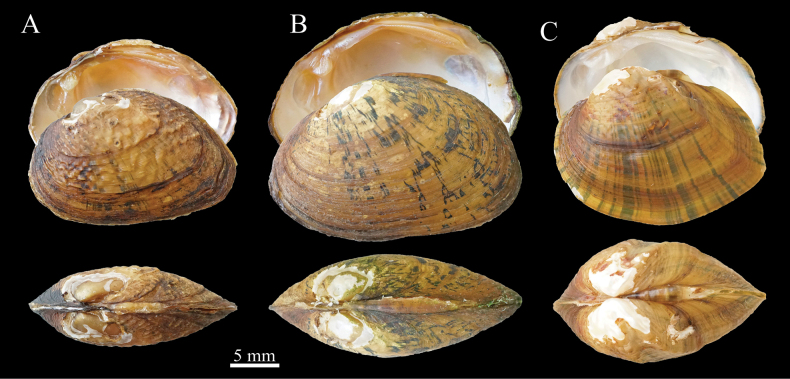
Specimens of *Diaurora* and *Acuticostachinensis***A***Diauroraaurorea*, 22_NCU_XPWU_DA03 **B***Diauroralaeve* sp. nov., holotype: 22_NCU_XPWU_DL01 **C***Acuticostachinensis*, 22_NCU_XPWU_AC01.

*Aurora* Simpson, 1900 was originally described as a subgenus of *Parreysia* based on shell morphology. Simpson (1900) indicated uncertainty regarding the taxonomic placement of *Unioauroreus* and whether it merited a generic rank. As a result, he provisionally designated it as a new subgenus. Cockerell (1903) renamed *Aurora* as *Diaurora* due to the former being a junior homonym of *Aurora* Ragonot, 1887 (Insecta: Lepidoptera: Pyralidae) and *Aurora* Sollas, 1888 (Porifera: Demospongiae: Ancorinidae). Haas (1924) elevated *Diaurora* to an independent genus, but this viewpoint was not widely accepted. Wu et al. (1999) recombined *Parreysiaaurorea* as *Acuticostaaurorea* based on shell morphology and marsupium type. While *Diaurora* shares some similarities in shell morphology with *Acuticosta*, it can be differentiated from *Acuticosta* by its distinct shell shape and the absence of a posterior ridge (Fig. 3).

#### ﻿ Diaurora aurorea

Taxon classificationAnimaliaUnionidaUnionidae

(Heude, 1883)

7EDF281C-E53F-53A8-9C9E-7B1BB926B1A8

Figs 3A , 4A , 5A

##### Museum material.

***Holotype***: USNM472411, Ning-kouo hien [宁国县], Kien-té hien [建德县], China. NZMC FM00732, Yuanjiang River [沅江], Hunan Province [湖南省], China. SMF24000a–b, Yütanchiao [玉潭桥], Hunan [湖南], China, 28°15′7″N, 112°33′45″E.

##### New material.

22_NCU_XPWU_DA01–02, Fuhe River [抚河], Fuzhou City [抚州市], Jiangxi Province [江西省], China, 27°53′57″N, 116°34′03″E, collected by Zhong-Guang Chen, Yu-Ting Dai, Chen-Chen Jia & Ying-Ying Zhang in September 2022. 22_NCU_XPWU_DA03–15, Ganjiang River [赣江], Jian City [吉安市], Jiangxi Province [江西省], China, 27°12′39″N, 115°09′44″E, collected by Zhong-Guang Chen & Zheng-Jie Lou in November 2022. YHC0028001, Jiulong River [九龙江], Fujian Province [福建省], China, collected by Hao Yang in 2003.

##### Diagnosis.

Shell triangular-ovate; posterior margin obliquely arc-shaped. Periostracum with straight broken blackish-green rays. Zigzag sculpture presented on all over the shell surface.

##### Description.

**Figure 4.**
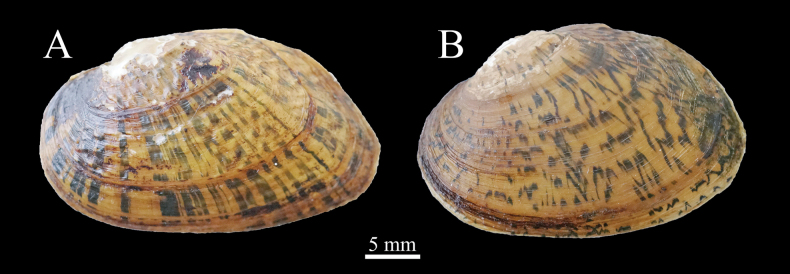
Comparison of the radial pattern on the shell surface in *Diaurora***A***Diauroraaurorea*, 22_NCU_XPWU_DA04 **B***Diauroralaeve* sp. nov., 22_NCU_XPWU_DL02.

Shell (Figs 3A, 4A). Shell small size, symmetric, solid, moderately thick, sub-glossy, triangular-ovate. Anterior margin oval, inflated; dorsal margin curved downwards; ventral margin slightly rounded or nearly straight; posterior margin obliquely arc-shaped. Umbo inflated, above hinge line, located at 1/3 of the dorsal margin, and often eroded. Periostracum orangish to brownish, with straight broken blackish-green rays and thin growth lines. Growth lines arranged in irregular concentric circles. Zigzag sculpture presented on all over the shell surface but weakening from umbo to edge. Hinge short. Ligament short and strong. Mantle muscle scars obvious. Anterior adductor muscle scars oval, deep, smooth in junior but rough in adult; posterior adductor muscle scars long oval, smooth. Left valve with two pseudocardinal teeth, equal height, anterior tooth small and flat, posterior tooth thick and pyramidal; anterior pseudocardinal tooth of the right valve well developed, posterior pseudocardinal tooth reduced, connected to lateral teeth. Lateral teeth of both valves long and thick. Nacre white or light orangish.

Length 32.0–41.3 mm, height 20.5–26.0 mm, width 12.4–17.2 mm.

**Figure 5.**
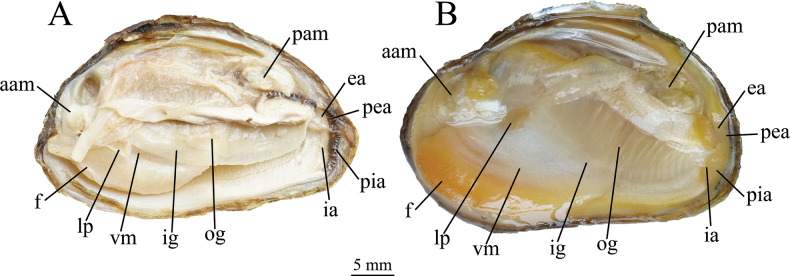
Soft anatomical characters of *Diaurora***A***Diauroraaurorea*, 22_NCU_XPWU_DA15 **B***Diauroralaeve* sp. nov., 22_NCU_XPWU_DL50. aam, anterior adductor muscle; pam, posterior adductor muscle; ea, excurrent aperture; ia, incurrent aperture; pia, papillae of the incurrent aperture; pea, papillae of the excurrent aperture; ig, inner gills; og, outer gills; m, mantle; lp, labial palps; vm, visceral mass; f, foot.

***Soft anatomy*** (Fig. 5A). Mantle off-white to light-brownish, aperture margins brown, flap margin with blackish to brown papillae. Gills light-brownish, inner gills slightly longer and wider than outer gills. Labial palps yellowish to brown (fade to grayish white in alcohol), distally pointed and irregularly fan-shaped in appearance. Visceral mass creamy white, foot orange (fade to grayish white in alcohol).

##### Vernacular name.

金黄蚌 (Pinyin: jin huang bang).

##### Distribution and ecology.

**Figure 6.**
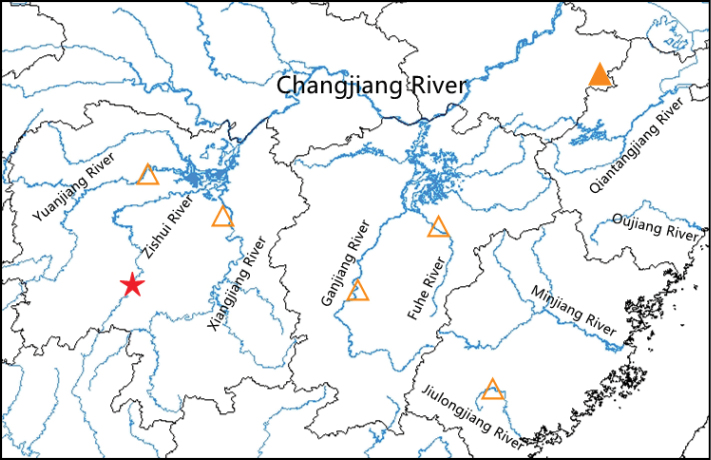
Distribution map of *Diaurora.* Star: type locality of *Diauroralaeve* sp. nov.; solid triangle: type locality of *Diauroraaurorea*; hollow triangle: new locality of *Diauroraaurorea*.

**Figure 7.**
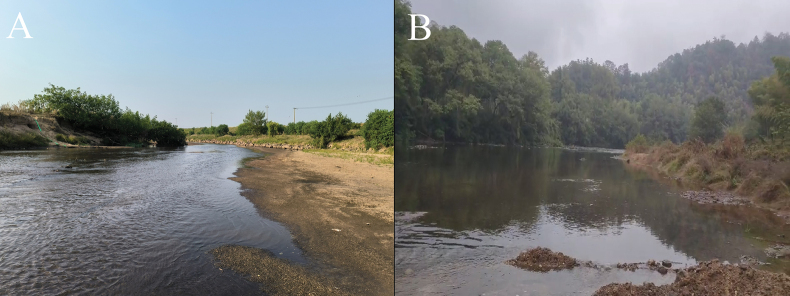
Habitat of *Diaurora.***A** habitat of *Diauroraaurorea*, Fuhe River, Fuzhou City, Jiangxi Province, China **B** habitat of *Diauroralaeve* sp. nov., Zishui River, Shaoyang City, Hunan Province, China.

Scattered in the middle and lower Changjiang River basin and the upper Jiulongjiang River, including Anhui Province, Jiangxi Province, Hunan Province and Fujian Province (Fig. 6). It mainly co-occurs with *Aculamprotulapolysticta* (Heude, 1877), *Acuticostachinensis* (Lea, 1868), *Cuneopsisheudei* (Heude, 1874), *Nodulariadouglasiae* (Gray, 1833), *Lamprotulacaveata* (Heude, 1877), *Lanceolariatriformis* (Heude, 1877) and *Ptychorhynchuspfisteri* (Heude, 1874) in the sandy substrates of tributaries of large rivers (Fig. 7). Besides, *D.aurorea* was reported in Vietnam by Dang et al. (1980), but the specimens were not available for examination (Do et al. 2018). This record is highly dubious due to its considerable distance from the known distribution range of *D.aurorea* as documented in this study. It is possible that this record was a misidentification of *Acuticosta* species.

##### Remarks.

The rarity of *D.aurorea* has been seriously underestimated for a long time due to the common misidentification of museum material. Upon examination of museum specimens, it has been discovered that specimen NZMC10542 in the National Zoological Museum of China and specimens 15_NCU_XPWU_AA01–11 in Nanchang University were actually *Acuticostachinensis*. Since its original description, only a handful of additional specimens of *D.aurorea* have been correctly identified. It has always been shrouded in mystery as there are hardly any photographs of specimens other than the type specimen. The new specimens examined in this study indicate that it is far more widespread than previously recorded. However, the habitats of *D.aurorea* are severely fragmented and the population size is very small. Therefore, it requires more attention and protection in the future.

#### ﻿ Diaurora laeve

Taxon classificationAnimaliaUnionidaUnionidae

Chen, Dai, Huang & Wu sp. nov.

2FAD2044-3B5E-503A-B354-9B0EC766F841

https://zoobank.org/06206EEC-D7DB-464E-AC35-56F9E9174000

Figs 3B , 4B , 5B

##### Type material.

***Holotype***: 22_NCU_XPWU_DL01, Zishui River [资水], Shaoyang County [邵阳县], Shaoyang City [邵阳市], Hunan Province [湖南省], China, 26°59′27″N, 111°16′10″E, collected by Zhong-Guang Chen & Zheng-Jie Lou in November 2022; ***Paratypes***: 22_NCU_XPWU_DL02–100, other information same as holotype.

##### Diagnosis.

Shell reniform. Periostracum with irregular broken blackish-green rays. Zigzag sculpture only presented in umbo area.

##### Description.

Shell (Figs 3B, 4B). Shell small size, symmetric, solid, moderately thick, sub-glossy, reniform. Anterior margin oval, inflated; dorsal margin curved downwards and truncate; ventral margin slightly rounded or nearly straight; posterior margin oval. Umbo inflated, above hinge line, located at 1/3 of the dorsal margin, and often eroded. Periostracum orangish to brownish, with irregular broken blackish-green rays and thin growth lines. Growth lines arranged in irregular concentric circles. Zigzag sculpture only presented in umbo area. Hinge short. Ligament short and strong. Mantle muscle scars obvious. Anterior adductor muscle scars oval, deep, smooth in junior but rough in adult; posterior adductor muscle scars long oval, smooth. Left valve with two pseudocardinal teeth, equal height, anterior tooth small and flat, posterior tooth thick and pyramidal; anterior pseudocardinal tooth of the right valve well developed, posterior pseudocardinal tooth reduced, connected to lateral teeth. Lateral teeth of both valves long and thick. Nacre light orangish.

***Holotype***: length 41.8 mm, height 29.1 mm, width 17.0 mm; Paratypes: length 27.4–45.0 mm, height 17.6–30.8 mm, width 11.0–17.6 mm.

***Soft anatomy*** (Fig. 5B). Mantle off-white to light-brownish, aperture margins brown, flap margin with yellowish papillae. Gills light-brownish, inner gills slightly longer and wider than outer gills. Labial palps yellowish to brown, distally pointed and irregularly fan-shaped in appearance. Visceral mass creamy white, foot orange.

##### Etymology.

The specific name *laeve* is made from the Latin *laeve* for smooth, an adjective, alluding to the smoother shell surface of this species.

##### Vernacular name.

平滑金黄蚌 (Pinyin: ping hua jin huang bang).

##### Distribution and ecology.

*Diauroralaeve* sp. nov. is known from the type locality only (Fig. 6). It was found to occur in a pebbly substrate of the river together with *Nodulariadouglasiae* and *Lanceolariatriformis* (Fig. 7). It is the dominant species in the habitat, accounting for 98% of the total density of freshwater mussels.

##### Remarks.

The placement of the new species in *Diaurora* is supported by both morphology and molecular phylogenetic analysis. *Diauroralaeve* sp. nov. can be easily distinguished from *D.aurorea* by its reniform shell, smaller zigzag sculptured area, and irregular rays on the shell. The different habitat preferences of *Diauroralaeve* sp. nov. and *D.aurorea* may have led to their differentiation. *Diauroraaurorea* is commonly found in the middle reaches of sandy substrate tributaries, while *Diauroralaeve* sp. nov. prefers to inhabit the upper reaches of pebbly substrate tributaries. In recent years, with more in-depth investigations and field surveys, new freshwater mussel species such as *Inversidensrentianensis* Wu & Wu, 2021 and *Pseudocuneopsissichuanensis* Huang, Dai, Chen & Wu, 2022 have been discovered in small tributaries of China (Wu et al. 2021; Wu et al. 2022). These findings suggest that the diversity of freshwater mussels in small tributaries of China remains to be fully explored, and future extensive exploration may lead to the discovery of other yet-to-be-described species.

## Supplementary Material

XML Treatment for Diaurora

XML Treatment for Diaurora aurorea

XML Treatment for Diaurora laeve

## ﻿Additional information

### Author ORCIDs

Zhong-Guang Chen https://orcid.org/0000-0003-2689-3321

Yu-Ting Dai https://orcid.org/0009-0001-5856-3987

Xiao-Chen Huang https://orcid.org/0000-0002-0242-3571

Xiao-Ping Wu https://orcid.org/0000-0002-8037-5640

### Data availability

All of the data that support the findings of this study are available in the main text.
